# Perceived Roles and Barriers in Delivering Community-Based Care: A Qualitative Study of Health and Social Care Professionals

**DOI:** 10.5334/ijic.7617

**Published:** 2023-10-18

**Authors:** Lixia Ge, Wan Fen Yip, Andy Ho Hau Yan, Eric Chua Siang Seng, Christina Chieh Pann Pei, Ian Leong Yi Onn, Evon Chua Yiwen, Sinma Tham, Ringo Ho Moon-Ho, Woan Shin Tan

**Affiliations:** 1 Health Services and Outcomes Research, National Healthcare Group Pte Ltd, SG; 2 School of Social Sciences, Nanyang Technological University, SG; 3 Lee Kong Chian School of Medicine, Nanyang Technological University, SG; 4 Continuing and Community Care, Tan Tock Seng Hospital, SG; 5 Population Health & Community Transformation, Yishun Health, SG; 6 Office of Community Integration, Woodlands Health Campus, SG

**Keywords:** qualitative study, community-based care, integrated care, health adversity, focus group discussions

## Abstract

**Introduction:**

As healthcare systems increasingly embrace population health management, the integration of health and social care to improve the health and well-being of individuals is crucial. Thus, we conducted a qualitative study in Singapore to understand health and social care professionals’ (HCPs and SCPs) perception of the roles they played in delivering community-based care.

**Methods:**

A descriptive phenomenological research design was adopted. HCPs and SCPs (n = 53) providing services in community settings were recruited purposefully and interviewed through eleven focus group discussions. Each session was recorded and transcribed. Thematic analysis was applied.

**Results:**

Our results revealed eight themes in three main categories describing the roles played by HCPs and SCPs, including: (1) delivering needs-based care in community settings; (2) activating and empowering clients in health care, and (3) fostering community-based sustainable support networks. Six barriers encountered while performing these roles were also identified.

**Discussion and Conclusion:**

Our results highlight that the roles of HCPs and SCPs go beyond the provision of direct medical and social care. They were involved in activating and empowering clients to take care of their health, and importantly, fostering community-based sustainable support networks to better empower individuals in coping with health challenges. The identified barriers shed light on areas for potential improvements for integrated community care.

## Introduction

As the population ages, the prevalence of multi-morbidity, disabilities, and dysfunctions is expected to rise [1]. The recognition of the unsustainability of hospital-centric care delivery system in meeting the increased care needs [2] has prompted the development of new community-based models of care in many countries and the adoption of community-oriented population health management strategies.

In Singapore, the drive to establish sustainable care services has led to multiple policy initiatives aimed at transitioning care from hospitals to the community and home settings [3]. This transformation promotes medical-social integration in the community and underscores the heightened responsibilities placed upon community-based health care and social care professionals (HCPs and SCPs). Their role now extends to providing holistic person-centric care in informal care settings [4]. This expanded role encompasses diverse expectations, including the implementation of timely and effective care transitions across settings, the delivery of personalized and needs-oriented support, and the facilitation of seamless coordination and collaboration among various care providers. Concurrently, within the landscape of population health management, policymakers are keen to harness existing community resources for the development of public health strategies and programmes. Hence, HCPs and SCPs are also tasked with nurturing the well-being of individuals, enhancing their resilience to prevent, cope with, and rise above health-related adversities, contributing to the creation of community-based assets to mitigate the negative impact of poor health. Nonetheless, achieving these goals requires a better understanding of the roles and efforts undertaken by community-based HCPs and SCPs, as well as understanding the challenges they confronted.

Published studies have examined the organisational roles and functions of HCPs and SCPs in integrated health-social care settings [5,6,7,8]. However, clear descriptions and documentation of their roles in delivering community-based care in Singapore are scarce. Gaining insights into the experiences of these frontline providers will provide insights to help policymakers identify service provision gaps and potential areas for improvement in population health initiatives. Furthermore, this knowledge can guide the creation of supportive measures to facilitate the community-based care delivery, such as the development of well-defined job scopes and tailoring on-job training and education programmes for community care providers.

In addition, literature has pointed out that culture influences population health needs, care seeking patterns, and care delivery [9,10]. As such, insights from western countries may not be directly applicable to Singapore. Hence, we conducted this qualitative study to better understand how community-based HCPs and SCPs in Singapore perceived their roles and to identify the barriers they confronted while providing community-based health and social care. Our research questions were:

1. How do HCPs and SCPs perceive their roles in the provision of community-based health and social care to support individuals in poor health?
2. What barriers did they encounter while fulfilling their roles?

## Methods

This qualitative study is part of a larger scale study aiming to develop and validate a health resilience measure in community-dwelling adults. The study employed a descriptive phenomenological approach [11] to explore and describe the perceived roles of community-based HCPs and SCPs in providing community care and the barriers or issues they encountered while fulfilling these roles.

### Study participants and sampling method

Participants were purposively recruited through three healthcare institutions located in the northern and central regions of Singapore. Managers who were responsible for the operations of community-based programmes assisted in sharing the study invitation letter with potential participants and referred HCPs and SCPs in any of the three healthcare institutions or their community partners in the central or northern regions if his/her work involved caring for the health and/or wellness of residents in the community.

Of the 83 referrals received, 9 were excluded as they were not involved in direct community care, 20 declined due to work commitment, and 1 withdrew after consenting due to family commitment. A total of 53 HCPs and SCPs (42 were from healthcare organisations and 11 were from social care organisations) who were involved in direct community care participated in the FGDs. Majority of the participants were aged between 21 – 40 years (n = 39, 73.6%), females (73.6%), and Chinese (86.8%).The HCPs were from the Community Health Team (or the equivalent team) in the three healthcare institutions which comprised an inter-professional team of nurses, allied health professionals, pharmacists, health coaches, operation staff, and medical social workers. They worked with SCPs and other professionals from their community partners in respective communities.

### Data collection

Data was collected via focus group discussions (FGDs). FGDs, which build on the group dynamics, allow for exploring diverse perspectives and enabling open and deeper discussions across various care providers working in health and social care organisations in the community setting, and allow researchers to reveal a range of viewpoints and insights that might not emerge through individual interviews [12].

To facilitate meaningful discussions, a semi-structured interview guide was developed to understand HCPs and SCPs’ perceived roles in community care. It contains a list of open-ended questions (Appendix 1) to guide and encourage participants to share their personal experiences and opinions on the research topic, which was incorporated with probing questions to delve deeper into participants’ responses, enabling a more comprehensive understanding of their perspectives. The interview guide was piloted to ensure that it elicited relevant and valuable information during the FGDs.

The FGDs were conducted virtually via a video conferencing platform (Zoom) between January and June 2021. Each FGD comprised a mix of four to six HCPs and SCPs from different organisations. This diverse composition allows for a wide range of perspectives relevant to the research topic were represented.

Each FGD was facilitated by an experienced female doctoral-level mixed-method researcher (TWS). The sessions were co-facilitated by two other female researchers, GLX who holds a master’s degree in public health, and YWF who is a doctoral-level epidemiologist. All researchers possessed substantial expertise in health services research and extensive experience in the healthcare sector. Throughout the discussions, the facilitators remained attentive to any potential dominance of certain participants or the halo effect, ensuring that all voices were heard, and that no individual overshadowed the others.

Each FGD lasted 90–120 minutes. To capture the valuable insights shared during these sessions, all FGDs were recorded using Zoom’s recording function with field notes taken. The audio recordings were transcribed into anonymised verbatim transcripts with a common format. The study team cross-checked the transcriptions against the original audio recordings to ensure the fidelity of the information without returning to participants for correction. SQR qualitative software NVivo version 12 [13] was used for coding, cross-referencing, storing, and retrieval of the data.

### Data analysis

As guided by the research questions, thematic analysis using an inductive approach, which primarily uses detailed readings of raw data to derive concepts or themes through researcher’s interpretations [14,15,16], was adopted to analyse the interview data. The procedures for the data analysis were outlined below.

1. Close reading of raw data: Two researchers (GLX and YWF) independently read the first three interview transcripts several times to familiarize with content and gain an understanding of key points and ideas mentioned by the participants.
2. Initial open coding: The two researchers openly coded the text line-by-line using specific or lower-level codes created from actual phrases or meanings in specific text segments and identified general or higher-level codes (themes) based on similarity and patterns observed in specific codes to address the research questions. Then they checked their sets of codes to establish the extent of overlap of the codes and themes.
3. Development of preliminary coding system: To ensure rigor, a discussion involving the facilitator (TWS) was held to review and establish a more robust set of emergent codes and themes. Consensus on the codes and themes were derived through multiple discussions among the three researchers. A working codebook was crafted and operational descriptions for the themes were created.
4. Continuing revision and refinement of the working codebook: The two researchers coded the remaining transcripts and searched for codes, including contradictory points of view and new insights. The codes and themes might be combined or linked under a superordinate category (summary category) when the meanings were similar. To address issues of trustworthiness and credibility, emergent themes were constantly compared and discussed. The refined codebook with examples of codes for each theme was presented in Appendix 2.

By employing these steps, the research team ensured the transparency of the thematic analysis. All the researchers maintained a neutral standpoint on the topic and practiced reflexivity throughout the research process [17]. We adopted the reporting style used by Williams and Irurita [18] to report findings for each theme.

### Ethical considerations

Ethical approval for this study was obtained from the National Healthcare Group’s Domain Specific Review Board (Reference number: 2019/00753). The study was conducted in accordance with the Declaration of Helsinki. Written informed consent was obtained from each participant prior to the commencement of the study.

## Results

Our analysis revealed 8 themes, which were further organised into three overarching summative categories that described the self-perceived roles of HCPs and SCPs in the provision of community-based health and social care to support individuals in poor health. The three categories were: (1) delivering needs-based community care (three themes); (2) empowering clients in managing their own health (two themes), and (3) building community-based sustainable support networks (three themes). Within each category, barriers to role fulfilment have also been identified.

### Category 1: Delivering needs-based community care

This category encompasses three distinct themes that illustrate the pivotal role played by HCPs and SCPs in providing direct and personalized care to address individuals’ needs in the community.

#### Theme 1: Providing direct medical care with continuity

Dedicated HCPs and SCPs played a pivotal role in delivering continued medical care, either during home visits or at respective Community Nursing Posts to ensure smooth care transition and good recovery progress, and to maintain their independence in the community. The types of medical care mainly included managing chronic conditions, reviewing laboratory test results, administering medications, improving independency in activities of daily living, providing and referring to secondary or tertiary care whenever needed (See Quote 1, Table 1).

**Table 1:** Quotes for themes and barriers identified in Category 1.


QUOTES FOR THE THEMES	QUOTES FOR THE BARRIERS IDENTIFIED

Theme 1: Providing direct medical care Quote 1: *“I do have a kind of diabetes programme with the patients. So, on a weekly basis, they come back to me to show me their blood glucose reading and also take photos of the food that they actually ate three times a week…and also they will share with me what are his activities for this past week.”* (Pharmacist, female) Quote 2: *“…one thing that will impact a lot is also how things are communicated [to] the patients and their family members…break down in very layman term, allowing the families to actually understand.”* (Medical Social Worker, female) Theme 2: Providing psycho-emotional support Quote 3: *“A lot of time I also feel that I kind of [providing] emotional support for them. Because at times they are lost, they don’t know what to do, we do give encouraging words for them to move on, and we also try to let them see what are their values in life, what are the things that they look forward to.”*(Nurse, female) Quote 4: *“I think apart from the eyes and ears, I think it’s really the human touch of the community partners that make the difference in their lives…So, I think all these community partners can act as a connection and their presence is their present.”* (Medical Social Worker, female)	Barrier 1: Being task-oriented instead of person-centred Quote 5: *“…we are pretty much task-focused. So, we go in, we want to do certain things for the patient and then, okay, that’s our duty done… a lot of times we forget to ask the patients or the residents there, what matters to them the most.”* (Allied Health Professional, female) Quote 6: *“Sometimes we fail to see the person as a whole… and we take them as…a person with this disease and we fail to recognize what their whole life was about…”*(Community Health Operation Officer, female) Barrier 2: Inadequate resources and manpower Quote 7: “*Family Service Centre can see you but they are so overworked, they are not going to see you very often. So, if you’re someone that is in real need of help, waiting for something more intense, you’re unlikely to get it*.” (Occupational Therapist, male)


HCPs and SCPs acknowledged the importance of good communication skills during care delivery. Hence, they gradually deployed better communication approaches (e.g., starting with understanding clients instead of being prescriptive, using layman terms instead of abstract medical terms, and identifying body cues) when talking to the clients so that the clients were more accepting of the message delivered (Quote 2).

#### Theme 2: Providing direct psycho-emotional support

HCPs and SCPs were aware that having hope and making necessary psychological adjustments in a person’s social roles or perception of life are essential parts of the process to thrive for better health. As some of the clients faced difficulties in accepting changes in their health and life, HCPs and SCPs provided psycho-emotional support to promote better psychological adjustments (Quote 3, Table 1). The types of psycho-emotional support included empathetic listening, providing space to talk and counselling services which assisted individuals in coping with emotional distress related to their health conditions (Quote 4).

While describing their roles of providing direct medical care and psycho-emotional support, participants emphasized the importance of showing respect and empathy and being sensitive to clients’ unmet needs, however, they acknowledged that sometimes they failed to see the person as a whole as they tended to be task-focused instead of person-centred while fulfilling their own KPIs and guidelines (See Quotes 5 and 6 for Barrier 1 in Table 1). Furthermore, constrained by limited resources and manpower, they faced challenges in offering care to all residents requiring assistance and providing care as frequently as necessary (Quote 7 for Barrier 2).

### Category 2: Activating and empowering clients in health care

Participants conveyed that their interactions with clients were often constrained by limited consultation time. This prompted HCPs and SCPs to underscore the significance of involving clients as active partners in their journey to surmount health challenges and recuperate from illness. The three themes encapsulated within this category illuminated the proactive approaches embraced by HCPs and SCPs to activate and empower clients, encouraging them to embrace a proactive role in taking charge of their health and well-being.

#### Theme 3: Reinforcing ownership of health

Participants acknowledged that the pivotal role that the application of self-management knowledge and skills plays in driving beneficial changes to their clients’ health and quality of life. Thus, their focus revolved around cultivating a sense of ownership among clients concerning their health (Quote 8 in Table 2). This involved fostering goal setting and implementing plans to support behavioural changes and the management of health conditions (Quote 9 in Table 2).

**Table 2:** Quotes for themes and barriers identified in Category 2.


Theme 3: Reinforcing ownership of health Quote 8: “*there’s this need to actually reinforce to them that they have to take ownership over…what’s going to happen next. Like be it their rehabilitation journey or …taking ownership of their own chronic diseases.”* (Physiotherapist, female) Quote 9: “*They have the information, they have seen us and educated them. But somehow, we still need something to hit them so that they start changing their lifestyle. They start bouncing back from their health crisis. So, there’s another group of patients who need something else in their lives to help them bounce back*.” (Dietitian, female) Theme 4: Imparting self-management knowledge and skills Quote 10: *“I think as a healthcare professional, our role is actually to provide the correct information and provide resources for them… a lot of times, our touchpoint is very short, so it’s really important to actually educate the patients or family [on] how to seek help correctly, then how to go through the difficult period and how to do the maintenance outside their own home. I think that’s our main role.”* (Community Nurse, female) Quote 11: “*I think information is a lot when you are dealing with a new disease or health adversity and it’s like learning a new topic…a new language around this whole issue… I think the ability for her [a client] to be able to do all these information sorting, not just gathering, helped her to move on to the next step because [if] she knew what to do next, she wouldn’t confused*. ” (Population Health Manager, female) Theme 5: Being navigators of community-based services and resources Quote 12: *“…financial wise we can bring in our medical social workers and for the practical resource we can bring in the home personal care, Meals on Wheels, etc., because we are very knowledgeable in what are the community resources out there … we can mobilize to help these family members because if they are on their own then they really would not know that there are such resources. So we do that form of guidance to navigate the resources that are out there.”* (Nurse, female) Quote 13: “…*update them of the resources that are available and also linking them up to the resources and also making sure that they are taken care of…*” (Nurse, male)	Barrier 3: Limited interaction time Quote 14: “*there are a lot of limitations in terms of time constraint. I only have 20 minutes with the patient and then I have to somehow motivate them within 5 minutes to do exercise. So, a lot of times, the only reason that I can give that allows me to get them moving is to say, “You want to get home soon? So, once you do your exercise, you can get well, we can get you home.” So, it kind of works for more than half the patients…this very short-term goal just to get home. But I do wonder at the back of their minds whether this goal is actually what they want in terms of their health and whether there are other things that can motivate them in terms of their outlook of life and how they can continue to live their years even outside of the hospital.”* (Occupational Therapist, female) Barrier 4: Restriction of eligibility criteria for financial assistance Quote 15: “*Like for example, somebody who has a spinal cord injury and then he’s on a ventilator. So, needs a lot, a lot of consumables, the expenses are actually a lot, which is probably way more than the kind of money that the whole family brings in. But because their salary income is already in the higher tier, so ultimately, they are not part of the financial assessment tier already. So, this kind of people will find a lot of difficulty in getting help.”* (Occupational Therapist, female)


However, participants pointed out that the pursuit of their role’s goal faced a significant challenge due to the constrained timeframe for consultations as they were not able to have a better understanding of clients’ life goals (See Quote 14 for Barrier 3 in Table 2). This limitation in interaction time with clients hindered their ability to facilitate in-depth discussions to motivate them in their own health management.

#### Theme 4: Imparting self-management knowledge and skills

Recognizing the significance of sufficient and reliable information for informed decision-making and self-health management, participants stressed the importance of providing clients with comprehensive resources. This encompassed not only general health knowledge and disease-specific information, but also extended to self-management knowledge and skills, as well as existing community-based resources. HCPs and SCPs perceived themselves as invaluable resource persons offering relevant and accurate information and imparting self-management through informal consultation, health coaching, and training, which were delivered during home visits and opportunistically at designated community-based wellness centres (Quotes 10 in Table 2). While fulfilling this role, participants were aware of the importance of balancing the amount of information provided against the individual’s ability to process it. Participants highlighted that to play an effective role in information provision, they must account for an individual’s actual understanding, information sorting, and navigation abilities (Quote 11 in Table 2).

#### Theme 5: Being navigators of community-based services and resources

The recognition of seamless access to services and resources in community as a critical enabler for self-health management underpins the significance of the role of HCPs and SCPs as navigators of community-based services and resources. While there is a myriad of community-based services, participants were aware of clients’ limited knowledge concerning the breadth of options and which specific services could address their unique care needs. Hence, community-based HCPs and SCPs empowered clients by aiding them in identifying and connecting with the appropriate services and community resources based on individual’s needs. The potential services and resources encompassed, but not confined to, financial support schemes, community health programs (e.g., the Wellness Kampung), and services provided by the community partners such as the Singapore Association for Counselling and the Family Service Centres (Quotes 12 and 13 in Table 2).

However, Participants underscored that financial assistance they could provide was generally basic and for the short-term. Due to the stringent eligibility criteria of existing financial assistance schemes or due to dignity concern, many clients were unable to receive any financial support despite high out-of-pocket heath care expenses (Quote 15 for Barrier 4 in Table 2).

### Category 3: Fostering sustainable community support networks

HCPs and SCPs held a common view that their role is to ultimately create a self-reliant and sustainable support system in community where residents do not have to rely on health and social care systems. To achieve this goal, HCPs and SCPs took an active stance to advocate for empowerment, grow the assets, and nurture the community’s intrinsic strengths. Within this category, three themes demonstrated their collaborative efforts in fostering community-driven sustainable support networks at multiple levels.

#### Theme 6: Strengthening family support

Participants emphasized the importance of family dynamics and relationship in the journey of recovery from health crises. They also were aware of the challenges and stresses faced by caregivers, particularly those attending to stroke patients with disabilities or dementia patients with behavioural issues. Hence, they intervened to harmonize family relationships and alleviate conflicts that hindered their clients’ recovery (Quote 16 in Table 3), provided situation-based support to caregivers for managing the demanding caregiving responsibilities and associated stressors, and equipped caregivers with caregiving knowledge and skills to strengthen family support (Quote 17 in Table 3).

**Table 3:** Quotes for themes and barriers identified in Category 3.


Theme 6: Strengthening family support Quote 16: “*Because if they are fighting at home, then…like the first thing is to kind of stop the fight. And then… it’s just really helping to link the pieces together and help everybody to be on the same page and like, “Okay, we’re starting at the same point. This is what your dad means. Okay, your daughter didn’t mean that but this is what she actually means.” and things like that.”* (Allied Health Professional, female) Quote 17: *“…he [an alcoholic man] wanted to get the support from the family members… So, we start to do some family dynamic to approach one by one and encourage them to have a communication in a way that maybe “he needs you”. Actually patient himself also requests that, “If a family member starts to motivate me to quit drinking, I’m more willingly to quit.” So, we encourage the elder son to participate in the conversation…I can see his condition become much more better. The amount of alcohol he drinks is gradually reduced compared to last time…his physical well-being was definitely better than before.”* (Nurse, male) Theme 7: Cultivating collaborative inter-organisational bonds Quote 18: “*For our Family Service Centre, we work very closely with a hospital. I think they have also extensively reached out to us…at different level… we start to go out together to do home visits to our clients to reach out to work on challenging cases. And also their MSWs and our social workers are also meeting for case conferences. We are also trying to bring in…tele health check in our centres. So, I think there’s a lot more collaboration [at] different levels*.” (Social Worker, male) *Quote 19: “We need to look at the bridge…And how can we shape this bridge in a way that it becomes more accessible for the community member to be able to cross in-between instead of making them run to five different places.”* (Manager of a social care organisation, female) Theme 8: Initiating community-based support collectives Quote 20: *“we’re thinking of building this community that is resilient to [be] able to thrive, able to manage with their own internal community support. So, to do that, I think we have to build the community. Not by us, but the community builds the community itself. We’ll just be facilitators…now we have this local area coordinators that we’re putting into the communities. So, through these local coordinators… we hope that we can help to pull the people together so that the community will be able to flourish and go into community building where they are able to provide the support needed within the community*. (Population Health Director, female) Quote 21: “*… the thing that we’re advocating and teaching and like trying to bring more people in is asset-based community development…so I would definitely advocate for us taking a backseat as a healthcare worker and really just creating and providing the opportunities for the communities to rise up to what they can do. So, [it] diminishes our presence eventually and get ourselves out of their lives as long as you know that they can be independent rather than creating reliance on [the] system and resulting in more workload for us.”* (Population Health Manager, female)	Barrier 5: Lack of a centralised system and streamlined integration Quote 22: *“I think we need to have a common platform whereby we are able to communicate so [as] to provide more seamless care for patients. And also, having said that, the patient also [have] clearer picture like who’s in the picture, who is actually doing what for me.”* (Nurse, male) Quote 23: *“Not very systematic and… there’s no central coordination or case management in the community for such things. Like for example, I can have a patient who requires several services… we probably don’t know each other is actually helping the patient… we don’t share the same system…a lot of things are blinded from the provider’s side… So, currently the systems are still quite…fragmented in a way.”* (Allied Health Professional, female) Barrier 6: Tendency of a more self-focused orientation Quote 24: *“I think it’s the Singapore culture, with the new and younger generation, that might block some of these from being organic developing on its own.”* (Medical Social Worker, female)


#### Theme 7: Cultivating collaborative inter-organisational bonds

Participants highlighted that they do not function in isolation in their clients’ recovery journey while providing care, instead, they collaborate with other care providers and partners at multiple levels and play a collective role according to the shift in care priority to better support their clients. To provide seamless integrated care, HCPs and SCPs within healthcare organisations embraced a proactive outreach to various community partners bi-directionally for consultation, and to bounce off ideas and to engage in collaborative decision-making and collective care delivery (See Quote 18 in Table 3). They also worked together to find solutions to better shape the “bridge” between health and social care providers to enhance service accessibility for the community residents (Quote 19 in Table 3).

Participants highlighted the issue regarding a lack of a centralised system and platform for patient information sharing and care coordination (Quote 22) and lack of a systematic streamlined integration (Quote 23 for Barrier 5 in Table 3). These caused ambiguity of client ownership, introducing problems in bringing together various elements of care, and hindering the efficient delivery of integrated services.

#### Theme 8: Initiating community-based support collectives

HCPs and SCPs described that they worked as the kick-starters to initiate the formation of community-based support groups which involve volunteers from different social groups. By doing so, they were creating opportunities to enable the community to contribute towards the building of sustainable community support networks (Quote 20 in Table 3). Participants were actively involved in advocacy work, identifying and harnessing existing community assets, fostering good-neighbourliness, and nurturing the very essence that allows communities to flourish (Quote 21 in Table 3).

Participants also noted the challenges inherent in advocating for the cultivation of strong community bonds as the younger generation of Singaporeans exhibited a more self-focused orientation, with a heightened emphasis on personal privacy (Quote 24 for Barrier 6 in Table 3). This tendency could potentially impede the natural growth of grassroots communities.

## Discussion

This study provided a comprehensive view of the roles played by HCPs and SCPs and the barriers they confronted in providing care in community-based settings. To our knowledge, this is the first qualitative study exploring their self-perceived roles and barriers in population health management spanning health and social care providers in the community settings in the context of Singapore. Their roles, which were described in eight themes and grouped into three overarching categories, have moved beyond traditional care provision and information dissemination [19] and expanded to perform broader functions within the integrated community care context. This expansion resonates with prior literature that has similarly highlighted the evolving functions of HCPs and SCPs in integrated settings [20,21].

In Singapore, shifting care from hospitals to community and strengthening residents’ self-management abilities have been put forth as strategies for bolster the sustainability of health system through reducing our reliance on tertiary care [3,22,23]. In this context, HCPs and SCPs are increasingly assuming roles in the community. Our study highlighted that they go beyond delivering direct care in community settings. By building rapport with community dwellers, community-based HCPs and SCPs gain profound insights into their clients’ life circumstances. This enabled them to construct comprehensive and coordinated care plans to meet individual client’s care needs, and to ensure a secure and seamless transition from hospital to community environments. Nonetheless, operating in the community settings is a relatively uncommon practice among HCPs and places more pressures to address unmet psychosocial needs of their clients that might go beyond their conventional scope of work. Hence, greater clarity about job expectations at the organisational level is required.

While care providers play significant roles in care provision, we found that HCPs and SCPs play crucial roles to encourage and enable individuals to be proactive in managing their own health [24] by imparting self-management knowledge and skills. Recent research has shown the protective influence of patient activation, engagement, and empowerment, which helps to reduce anxiety, stress and health-related uncertainty [25,26,27], and shapes positive coping behaviours [28]. Yet, in practice, akin to prior studies [29,30], the challenges of insufficient resources and time constraints thwart the implementation of evidence-based techniques by HCPs and SCPs in their pursuit of activating and empowering individuals. These practical barriers warrant diligent attention to ensure the realisation of more robust outcomes of integrated care.

Extensive evidence have shown that well-networked and supportive communities have a positive impact on people’s health and wellbeing [31]. Building sustainable support networks requires active involvement of assets within communities (e.g., people, knowledge and skills, social networks and community organisations). Healthcare and social care organisations are important players in improving health and reducing inequalities within communities [31]. HCPs and SCPs in this study highlighted that they have been fostering community-based sustainable support networks through multi-level efforts, starting with strengthening family support, cultivating collaborative inter-organisational bonds at the meso level, and initiating community-based support collectives at the society level.

In the Singaporean society, the cherished values of filial piety, encompassing familial care responsibilities, and the revered “kampong spirit”, representing a spirit of neighbourly connection and mutual support, hold considerable significance. Within this cultural context, families, neighbours and volunteers are regarded as the main informal pillars of strength and support [32] and play a pivotal role in the sustainability of the community support networks. To better support individuals and communities, building collaborative linkages across care providers to transcend conventional institutional boundaries will pool efforts toward a common objective of improved population health. As it is highly unsustainable to only rely on formal care providers [4], HCPs and SCPs play an important role in linking formal institutions with informal support groups (e.g., families and volunteers) to supplement or even substitute the roles of HCPs and SCPs.

### Practice implications

This study demonstrates the roles played by HCPs and SCPs in delivering care to community-dwelling adults. The challenges and barriers identified in the study (e.g., being task-oriented, inadequate resources and manpower, lack of a centralised system and streamlined integration, and the issue of the ownership of clients) reflect the current situation of community care in Singapore and provide some insights for the review of the integration process and the sustainability of current integrated care model. Collaborative efforts across healthcare and social care sectors to improve the breadth and depth of the integration (e.g., building an efficient information exchange platform, redefining ownership) are required to better address these issues. The newly launched PACE-It mobile app [33] – a secured chat platform allows users of multidisciplinary care team to contribute to and share data and the practice adopted by the Integrated Community of Care pilots [34] are good strategies. In addition, training more volunteers to become lay health workers [35] to provide care support in communities might be a sustainable alternative to address the sustainability issue.

### Strengths and limitations

The study has several strengths. First, both HCPs and SCPs across different position levels from various organisations were recruited and shared freely during the discussion. Hence, the findings of the study should be able to represent the actual roles performed by community-based HCPs and SCPs. Second, the systematic processes of inquiry and data analysis enable the study team to capture a comprehensive overview of HCPs and SCPs roles which were inductively emerged from experiences and narratives. It is worth mentioning that our study might suffer from the potential biases inherent in FGDs, which mainly include 1) social desirability bias as participants may alter their responses to align with perceived social norms, 2) peer pressure as participants might be influenced by the prevailing opinions within the group, 3) moderator bias as the moderator’s tone, or personal beliefs might influence the direction of the discussion, and 4) selection bias as the HCPs and SCPs were informed of the study by the managers who were actively involved in population health management and community care transformation, and those who participated might have a higher interest in the research topic than those who did not participate. In addition, only HCPs and SCPs in one regional health system of Singapore were recruited, hence, the findings may not be generalizable to other regional health systems if there are differences in real practice and should be interpreted with caution. Further research should investigate whether the roles described are similar among community-based HCPs and SCPs in other regional health systems.

## Conclusion

Our results highlight that the roles of HCPs and SCPs go beyond community-based direct care provision. They were involved in activating and empowering clients in health care, and importantly, fostering community-based sustainable support networks to better support individuals in coping with health challenges.

## Additional Files

The additional files for this article can be found as follows:

10.5334/ijic.7617.s1 Appendix 1. FGD Interview guide.

10.5334/ijic.7617.s2 Appendix 2. The refined codebook with codes, themes and categories.
