# Supplementary material for: Perceived Roles and Barriers in Delivering Community-Based Care: A Qualitative Study of Health and Social Care Professionals
Source: Int J Integr Care. 2023 Oct 18;23(4):5. doi: 10.5334/ijic.7617 (PMC10591202; doi:10.5334/ijic.7617)
Supplement: Appendix 1. — FGD Interview guide. [file ijic-23-4-7617-s1.pdf]

1. What is your role as a healthcare / social care professional in supporting individuals to bounce back from an adverse health event?
   (*Probe: What did they need most and what was your role?)*

2. What is the role of community-based healthcare services and social service organisations in supporting them?

3. (We often hear that clients or patients may require services across health and social care providers) From your experience, how do clients or patients navigate across these services?

4. What is the role of social network (friends or neighbours) when faced with health adversities?
   (Probe: Who supported you?)
   (Probe: How did they help?)

5. What else can we do as a healthcare / social care system to better support individuals to bounce back from an adverse health event?

6. What else can we do as a society?
