# Supplementary material for: Perceived Roles and Barriers in Delivering Community-Based Care: A Qualitative Study of Health and Social Care Professionals
Source: Int J Integr Care. 2023 Oct 18;23(4):5. doi: 10.5334/ijic.7617 (PMC10591202; doi:10.5334/ijic.7617)
Supplement: Appendix 2. — The refined codebook with codes, themes and categories. [file ijic-23-4-7617-s2.pdf]

Appendix 2. The refined codebook with codes, themes, and categories

Examples of codes	Themes	Categories
• Ensuring care continuity<br>• Medication management<br>• Monitoring disease progress<br>• Personalized rehabilitation to regain physical function<br>• Providing medical advice<br>• Helping understanding of disease and treatment<br>• Providing holistic / tailored care with flexibility<br>• Referring to specific medical care services when needed<br>• Providing comfort care	Providing direct medical care	**Delivering needs-based care in community settings**
• Emotional support / counselling /reassurance<br>• Giving encouragement / affirmation<br>• Psycho education aligning patient's goals<br>• Finding / fostering hope<br>• Initiating reflection process<br>• Discussing life goals<br>• Listening and giving space to talk<br>• Proving human touch / being present<br>• Helping discover own strengths and resilience<br>• Promoting psychosocial adjustment /adaption<br>• Empathizing	Providing psycho-emotional support	
• Health counselling<br>• Reinforcing ownership of their own chronic diseases<br>• Initiating lifestyle changing<br>• Finding patients' motivation<br>• Initiating goal setting aligning with their values in life<br>• Motivational interviewing<br>• Partnering with clients	Reinforcing ownership of health	**Activating and empowering clients in health care**
• Providing health education / coaching / training<br>• Equipping coping skills<br>• Teaching exercise techniques<br>• Teaching disease/symptom management skills	Imparting self-management knowledge and skills	
• Needs-based social services<br>• Linking clients up with available resources<br>• Directing clients to appropriate community partners<br>• Being connectors<br>• Exploring possible options	Being navigators of community-based services and resources	
• Moderating expectations of clients and family<br>• Harmonizing family relationship<br>• Providing training/support to family caregivers<br>• Addressing caregiving burden	Strengthening family support	**Fostering community-based sustainable support networks**
• Playing a collective role for clients<br>• Consulting partners for decision making<br>• Working together to support a common care plan<br>• Interchangeable care leadership across partners<br>• More collaboration at different levels<br>• Knowledge and experience sharing	Cultivating collaborative inter-organisational bonds	
• Developing asset-based community with residents<br>• Providing opportunities for communities to come together<br>• Forming various support / interest groups /initiatives<br>• Harnessing "kampung/community spirit"<br>• Providing space for informal networking<br>• Engaging community enablers<br>• Coordinating volunteers' efforts	Initiating community-based support collectives	
